# Editorial Note: Detection of relapsing fever *Borrelia* spp., *Bartonella* spp. and Anaplasmataceae bacteria in argasid ticks in Algeria

**DOI:** 10.1371/journal.pntd.0014351

**Published:** 2026-05-22

**Authors:** The PLOS Neglected Tropical Diseases Editors

The *PLOS Neglected Tropical Diseases* Editors issue this notice to update the previously published Expression of Concern on this article [1,2].

Following the publication of the article and Expression of Concern [1,2], PLOS investigated concerns pertaining to the reported ethical approval and the article’s adherence to *PLOS Neglected Tropical Diseases*’ research ethics policies.

Specifically, the Materials and methods section of [1] reports that ticks were collected between May 2012 and October 2015. Furthermore, the article reports that the sampling locations involved a variety of natural and human-impacted habitats, and included rodent burrows in farms, yellow-legged gull nests (after the nesting period ended), and “other animal shelters,” located in Mostaganem, Algiers, El Tarf, and M’sil (Algeria). The Ethical considerations section of the article states that collection sites excluded national parks, protected areas, and endangered or protected species, and that owners of homes and private land provided permission for the sampling. Furthermore, the article reports that the study protocol was approved by the Steering Committee of the Algerian Ministry of Health (Direction Générale de la Prevention), but it does not cite a document reference number, and it does not report any field permits.

The corresponding author stated that this study was conducted in compliance with the applicable laws and regulations in Algeria and France, with the support of the relevant academic institutions in these countries, but the documentation they provided for editorial review was insufficient to fully resolve the journal's concerns.

A representative of the Aix-Marseille Université Ethics Committee stated that the institutional investigation into the ethics concerns concluded this article does not violate French regulations or international ethics rules. They provided the approval document #203/MZ/DGPPS/2017 for editorial review.

Document #203/MZ/DGPPS/2017 was issued on February 14, 2017 by the Direction Générale de la Prévention et de la Promotion de la Santé of Algeria, approving the object: “Surveillance entomologique dans vos déparments.”

PLOS reviewed the documentation provided by the institution and concluded that the documents did not fully resolve the concerns. Therefore, the Expression of Concern stands.
